# Children at the Risk of Recurrent Wheezing: A Matched Case-Control Study in a Tertiary Care Center

**DOI:** 10.7759/cureus.35387

**Published:** 2023-02-23

**Authors:** Halak Vasavada, Snehal Patel, Hetal Vora, Riya Agrawal, Krutik Gamit, Ruchi Pagi, Nirali Desai, Ravina Rakholiya, Krupa Modi

**Affiliations:** 1 Department of Paediatrics, Smt. Nathiba Hargovandas Lakhmichand Municipal Medical College, Ahmedabad, IND

**Keywords:** wheezing, wheezing in children, recurrent wheezing, exclusive breast feeding, significant association, matched controls, risk factors

## Abstract

Introduction

Wheezing is a common symptom in early childhood. Recurrent wheezing is defined as more than three episodes of wheezing in the past year. Many studies have been conducted to delineate the risk factors for recurrent wheezing and to predict which of these children will progress to asthma. Most studies about risk factors and the clinicodemographic profile of children with recurrent wheeze have been carried out in developed nations. Data in developing countries may differ. This study was carried out to identify risk factors associated with recurrent wheezing in children in a tertiary care center.

Materials and methods

It was a retrospective, matched case-control study conducted over a period of two years (July 2019 to July 2021). Records of children aged one month to 12 years who came to pediatric OPD or were admitted to a pediatric ward with a history of recurrent wheezing were included in the study. Cases with uncontrolled recurrent wheezing diagnosed by examination with an unreliable history and those with a global developmental delay were excluded from the study.

The study involved the hospital records of 60 children. Of these, 30 were recurrent wheezers, and 30 were non-wheezers (controls). Data were collected with detailed proformas from case histories and examination sheets. The proforma had several known and suspected risk factors associated with wheezes. Each risk factor was studied and compared with the control group. The risk factors included in this study were male gender, not exclusively breastfed, history of bottle feeding, exposure to vehicles; exposure to pollen; exposure to animals; using an agarbatti or dhoop, passive smoking, or playing with a soft toy. Data were entered in an Excel sheet, and appropriate statistical analyses were done.

Results

The male-to-female ratio was 2:1. Out of the number of cases, 73.33% were younger than six years; 56.66% of cases were not exclusively breastfed, and 43.33% were exclusively breastfed for six months; 20% of the cases were bottle-fed, and 40% of the controls were bottle-fed. The percentage of cases exposed to vehicle smoke was 26.66%, while 20% of cases had exposure to pollen and 16% of controls were exposed to pollen. 30% of cases were exposed to animals, and 23% of controls were exposed to animals. With regard to passive smoking, 16.66% of cases were exposed to passive smoking, and 20% of controls were not exposed to passive smoking. Out of the study group, 26.66% of the children played with soft toys. Of all these risk factors, a significant difference between cases and controls was found in only one factor: not being exclusively breastfed for six months. All other risk factors showed no significant difference between cases and controls.

Conclusion

The present study concluded that the significant risk factor that was associated with recurrent wheezing was "not exclusively breastfeeding." The other factors studied that were suspected to be associated with recurrent wheezing cannot be ruled out entirely due to the relatively small size of the sample and the need to be studied further in detail.

## Introduction

Wheezing can be described as a high-pitched sound characteristic of intra-thoracic obstruction, usually due to turbulent flow in narrow airways. Wheezing is a common symptom in early life, with almost 50% of children having at least one episode of wheezing in their first year of life [1]. Recurrent wheezing has been defined by the National Asthma Education and Prevention Program Expert Panel as "more than three episodes of wheezing in the past year that lasted more than one day and affected sleep" [2]. In young children, wheezing, either transient or persistent, can be severe and cause poor quality of life with frequent use of the health care system and economic costs. Recurrent wheezers have significant morbidity, and it is estimated that about one-third of school-age children manifest the symptom during the first 5 years of life. Children with wheezing have an increased risk of developing asthma and allergies, but not all children who wheeze have asthma.

For years, scientists have attempted to predict which children with recurrent wheezing are more likely to develop asthma at school age. Different factors-phenotypic and genotypic-have been proposed for precise prediction and individualized treatment plans. The main problem has been the under- or overestimation of characteristics or symptoms in children with recurrent wheezing. [3]

The Tucson Children’s Respiratory Study (TCRS) was begun in 1980 as a long-term longitudinal study to investigate the interrelationships between many potential risk factors, acute lower respiratory tract illnesses during the first three years of life, and the development of chronic lung disorders, especially asthma, in later childhood and young adult life [4]. However, the correlation between recurrent wheezing and the risk factors influencing it has not been exclusively documented. Considerable investigation has been directed at the long-term prognosis of early childhood wheeze risk factors for early wheeze development into asthma and the tools to predict which children will develop asthma. However, despite vast work in the area, the factors that influence recurrent wheezing remain complex and not well understood. Most of the studies regarding risk factors for recurrent wheezing have been conducted in developed nations, where the prevalence of asthma and allergies is high. The risk factor profile for recurrent wheezing in developing countries like India may significantly differ from that in developed nations. Therefore, this study aims to investigate the demographic profile and the risk factors associated with recurrent wheezing in children attending a tertiary care center.

## Materials and methods

The study was conducted at Shardaben Hospital, a tertiary care teaching hospital in Saraspur, Ahmedabad, India. Approval from the local authority was obtained for conducting the study, and informed consent was obtained. All the data was de-identified. It was a retrospective, matched case-control study involving hospital records taken over a period of two years (July 2019 to July 2021).

The inclusion and exclusion criteria are presented in Table 1.

**Table 1:** Inclusion and exclusion criteria

Inclusion criteria	Exclusion criteria
Case records of children one month to 12 years coming to pediatric OPD or being admitted to the pediatric ward with a history of recurrent wheeze	Uncontrolled recurrent wheeze by examination with an unreliable history
	Children with global developmental delay

Sampling technique

Methods of Data Collection

Hospital records of 30 children with a history of recurrent wheezing satisfying the inclusion and exclusion criteria and coming on an OPD basis or being admitted to the pediatric ward were taken up for study. Data were collected in the form of a detailed history and medical examination. The proforma was completed by taking a detailed history from records. The working definition of recurrent wheezing was three or more episodes of wheezing in the last year.

The risk factors that each child was exposed to were documented in a detailed proforma. They included exclusive breastfeeding, bottle feeding, exposure to vehicle smoke, exposure to animals, exposure to pollens, passive smoking, exposure to incense (agarbatti and dhoop), and playing with soft toys.

Thirty patients were taken from the indoor cases admitted for other conditions and placed in the control group for comparison. They were matched for age, gender, socioeconomic status, and place of healthcare visits. The date of admission for the control group was within two days of the date of admission for the cases. The indications for admission for the control group were non-respiratory. Similar data regarding risk factors was collected using records from the control group. Risk factors included exposure to vehicle smoke, which is considered positive when the patient lives in a traffic-prone area. Traffic-prone areas for Ahmedabad were defined based on criteria defined by the municipal authorities. Exposure to animals was considered a positive risk when the patient had a pet in the house or lived near a stable. Another risk factor was exposure to pollen, which was considered positive when the patient had plants with flowers in the house or lived near a garden.

Outcome Measures

All data were entered into an Excel sheet. Demographic variables were represented using percentages. A Chi-square test was applied for significant risk factor association using appropriate statistical software.

Limitations

Owing to the small sample size, the odds ratio was not used as a statistical measure. Logistic regression analysis also could not be done.

## Results

The present study included 30 cases and 30 controls who were matched for age and gender, that is, they had similar age and gender characteristics as the cases.

In the present study, as seen in Table 2, out of the 30 patients, 22 (73%) are under six years of age, and eight (27%) are over six years of age. This data suggests that recurrent wheezing is more common in children in the younger age group (under six years).

**Table 2:** Distribution of cases and controls according to age

Age	<6 years	>6 years	Total
Numbers	22	8	30
Percentage	73.33%	26.66%	100%

Out of the 30 patients, as shown in Table 3, 20 (66.66%) were male children and 10 (33.33%) were female children.

**Table 3:** Distribution of cases and controls according to gender

Gender	Male	Female	Total
Number	20	10	30
Percentage	66.66%	33.33%	100%

Table 4 shows that 56.66% of cases were not exclusively breastfed, while among controls this percentage was 23.33%. The p-value by Chi-square test at a 95% confidence interval (CI) was 0.008 (p<0.05) which suggests the absence of exclusive breastfeeding as a significant risk factor in children with recurrent wheezing as compared to age-matched controls.

The study also showed that bottle feeding was present in 20% of cases but not in 40% of controls, making the p-value 0.311 (not significant). Also, vehicular smoke exposure among cases and controls showed no significant difference (p 0.573), as did passive smoking (p 0.738). Exposure to pollen was observed in 20% of cases and 16.66% of controls, with a p-value that was insignificant. Exposure to incense was seen in 30% of cases and 46.6% of controls, yet the p-value fell short of the significance level (0.184). Playing with soft toys was found in eight cases (26.66%) and more in controls (40%); the difference, however, was not significant.

**Table 4:** Distribution of cases and controls according to risk factors

Risk factor	Group		Total	p-value
	Cases	Controls		0.008
Not exclusively breastfed for six months	17 (56.66%)	7 (23.33%)	24 (40%)	
Exclusively breastfed for six months	13 (43.33%)	23 (76.66%)	36 (60%)	
Bottle feeding	6 (20%)	12 (40%)	18 (30%)	0.311
No bottle feeding	24 (80%)	18 (60%)	42 (70%)	
Exposure to vehicle smoke	8 (26.66%)	10 (33.33%)	18 (30%)	0.573
No exposure to vehicle smoke	22 (73.33%)	20 (66.66%)	44 (70%)	
Exposure to pollen	6 (20%)	5 (16.66%)	11 (18.33%)	0.738
No exposure to pollen	24 (80%)	25 (83.33%)	49 (81.66%)	
Exposure to animals	9 (30%)	7 (23.33%)	16 (26.66%)	0.559
No exposure to animals	21 (70%)	23 (76.66%)	44 (73.33%)	
Passive smoking	5 (16.66%)	6 (20%)	11 (18.33%)	0.738
No exposure to passive smoking	25 (83.33%)	24 (80%)	49 (81.66%)	
Exposure to agarbatti or dhoop	9 (30%)	14 (46.66%)	23 (38.33%)	0.184
No exposure to agarbatti or dhoop	21 (70%)	16 (53.33%)	37 (61.66%)	
Playing with soft toys	8 (26.66%)	12 (40%)	24 (40%)	0.273
Not playing with soft toys	22 (73.33%)	18 (60%)	36 (60%)	

## Discussion

This study, conducted in the department of pediatrics at a tertiary care center, was a retrospective case-control study from July 2019 to July 2021 involving 60 children. Of these, 30 are recurrent wheezers, and 30 are non-wheezers (controls). Data were collected using a detailed proforma from hospital records. The proforma had several known and suspected risk factors associated with wheezes. Each known or suspected risk factor was studied and compared with the control group.

The risk factors studied were age, gender, not exclusively breastfed babies, bottle feeding, exposure to vehicle smoke, exposure to pollens, exposure to animals, passive smoking, exposure to incense (agarbatti or dhoop), and playing with soft toys. Out of these, the absence of exclusive breastfeeding was found to be a significant risk factor for recurrent wheezing in children.

The study showed that more than 70% of the children were under six years old. It is a known fact that children younger than two to three years are especially prone to wheezing because bronchospasm, mucosal edema, and the accumulation of excessive secretions have a relatively greater obstructive effect on their smaller airways. In addition, the compliant airways in young children collapse more readily with active expiration [2].

Similar results were found in a study by Klaita Srisingh et al., which suggests that the prevalence of wheezing decreases as age increases: 32% in the first year, 17.3% in the second year, and 12% in the third year [5].

In the present study, 66% of the children were male. The male gender is an important risk factor for recurrent wheezing [6,7]. A study by Bozakayut et al. showed 61% of the study population was male [8]. These results are also comparable to a study done by Nitin et al. in Uttar Pradesh on the prevalence and risk factors for recurrent wheezing in schoolchildren, which showed 55.9% of males and 44.1% of females were affected. In the study done by Roberta Barros, males were prevalent in the wheezing group (p = 0.041) [9]. However, there are studies that did not find any significant difference between males and females. [10]. Giulbert et al. found that male patients are more sensitive to andro-allergens [11].

In the present study, out of 30, 56.66% of patients were not exclusively breastfed, as opposed to 23.3% of controls who were not exclusively breastfed. The difference was significant, with a p-value of 0.008 at the 95% CI. Thus, non-exclusively breastfed children were more likely to have recurrent wheezing as compared to children under similar control.

Breastfeeding is protective against recurrent wheezing [12-15]. The findings of the present study are supported by Bozakayut et al., which showed an inverse relationship between the duration of breastfeeding and recurrent wheezing episodes, where the rate was 54% in infants breastfed for less than six months [8].

In contrast to the present study, Srisingh didn’t find an association between breastfeeding and recurrent wheezing (p=0.830) [4]. Taveras et al. also did not find any difference in terms of recurrent wheezing in children who were breastfed for more than nine months [16].

The study finds no significant difference in the occurrence of wheezing between children who are bottle-fed and those who are not. In contrast to this study, the Juan C. Celedon study shows that children who were bottle-fed in bed before sleep had a 1.5 times higher risk of wheezing between the ages of one and five years [17].

The present study finds no relation to exposure to vehicular smoke, as 26% of cases and 33% of controls were exposed to vehicular smoke. Roberta Barros' study [9] observes exposure to smoke caused by environmental pollution associated with wheezing (p=0.010). Also, a study by Singh et al. found an odds ratio (OR) of 1.63 (95% CI: 1.43, 1.85), 1.71 (95% CI: 1.49, 1.96), and 1.53 (95% CI: 1.31, 1.78) with mild, moderate, and heavy traffic pollution, respectively [18].

There is no statistically significant association found between cases of recurrent wheezing and exposure to animals. This may be because a smaller number of both cases and controls involved animal exposure. Many studies have documented an association between animal exposure and asthma. Hugg et al. demonstrated a significant risk of asthma in children exposed to dogs [19]. In the study by Abdulkadir Bozakayut [8], pets were found to be protective against recurrent wheezing (p=0.0001).

Passive smoking is traditionally considered a risk factor for recurrent wheezing. The present study did not find any significant association. Similar results were found in Roberta Barros' [9] study (p=0.130). Srisingh's [5] study shows passive smoking as a significant risk factor for wheezing (p 0.027). A study by S. Singh et al. also shows an OR of 1.21 (significant) in relation to recurrent wheezing and parental smoking.

Similarly, exposure to pollen, exposure to incense sticks (agarbatti or dhoop), and playing with soft toys were also not found to have a significant correlation with recurrent wheezing.

The strength of the study was the retrospective case-control design, which can give a comparative analysis of the effects of various risk factors for recurrent wheezing in children.

Limitations

A major limitation of the present study was the small sample size. Hence, the significance of other factors suspected to be associated with recurrent wheezing cannot be ruled out. A better study design and more extensive sampling would be needed to delineate the risk factors more accurately.

## Conclusions

The study concluded that recurrent wheezing is common among children under six years of age. It was also found to be more common in male children. Babies who were not exclusively breastfed up to six months of age had a higher prevalence of recurrent wheezing as compared to controls. Thus, breastfeeding was found to have a protective role in preventing recurrent wheezing.

Other known factors like bottle feeding, exposure to vehicle smoke, pollens, animals, passive smoking, incense sticks (agarbatti and dhoop), and playing with soft toys were not found to have a significant association with recurrent wheezing in the present study. Some previous studies have found significant associations with these risk factors, but there are other studies that have not found such associations. These other factors studied that were suspected to be associated with recurrent wheezing cannot be ruled out entirely due to the relatively small size of the sample and need to be studied further in detail with a more intensive and extensive study design.
